# Improving EEG-Based Emotion Classification Using Conditional Transfer Learning

**DOI:** 10.3389/fnhum.2017.00334

**Published:** 2017-06-27

**Authors:** Yuan-Pin Lin, Tzyy-Ping Jung

**Affiliations:** ^1^Institute of Medical Science and Technology, National Sun Yat-sen UniversityKaohsiung, Taiwan; ^2^Institute for Neural Computation, University of CaliforniaSan Diego, San Diego, CA, United States

**Keywords:** EEG, classification, emotion, music, individual difference, transfer learning

## Abstract

To overcome the individual differences, an accurate electroencephalogram (EEG)-based emotion-classification system requires a considerable amount of ecological calibration data for each individual, which is labor-intensive and time-consuming. Transfer learning (TL) has drawn increasing attention in the field of EEG signal mining in recent years. The TL leverages existing data collected from other people to build a model for a new individual with little calibration data. However, brute-force transfer to an individual (i.e., blindly leveraged the labeled data from others) may lead to a negative transfer that degrades performance rather than improving it. This study thus proposed a conditional TL (cTL) framework to facilitate a positive transfer (improving subject-specific performance without increasing the labeled data) for each individual. The cTL first assesses an individual’s transferability for positive transfer and then selectively leverages the data from others with comparable feature spaces. The empirical results showed that among 26 individuals, the proposed cTL framework identified 16 and 14 transferable individuals who could benefit from the data from others for emotion valence and arousal classification, respectively. These transferable individuals could then leverage the data from 18 and 12 individuals who had similar EEG signatures to attain maximal TL improvements in valence- and arousal-classification accuracy. The cTL improved the overall classification performance of 26 individuals by ~15% for valence categorization and ~12% for arousal counterpart, as compared to their default performance based solely on the subject-specific data. This study evidently demonstrated the feasibility of the proposed cTL framework for improving an individual’s default emotion-classification performance given a data repository. The cTL framework may shed light on the development of a robust emotion-classification model using fewer labeled subject-specific data toward a real-life affective brain-computer interface (ABCI).

## Introduction

Electroencephalogram (EEG)-based emotion classification has led to an emerging and challenging track in affective brain-computer interface (ABCI) domain (Mühl et al., 2014; Lin et al., 2015b). Referring to the prior studies (Chanel et al., 2009; Frantzidis et al., 2010; Lin et al., 2010b, 2014, 2015a; Petrantonakis and Hadjileontiadis, 2010; Koelstra et al., 2012; Soleymani et al., 2012; Hadjidimitriou and Hadjileontiadis, 2013; Koelstra and Patras, 2013; Jenke et al., 2014), a potential bottleneck of developing a practicable emotion-classification model for an individual could be the lack of using sufficient ecologically-valid data, especially for the works (Koelstra et al., 2012; Soleymani et al., 2012; Koelstra and Patras, 2013; Lin et al., 2015a) using long-duration emotion elicitation, such as movie watching and music listening. Specifically, the reported classification accuracies generally varied between 55% and 72% (Koelstra et al., 2012; Koelstra and Patras, 2013; Lin et al., 2015a) for a binary classification task and between 52% and 57% for a three-class task (Soleymani et al., 2012). The limited EEG trials pose a significant barrier to encompass the EEG tempo-spectral dynamics associated with implicit emotional responses for an individual.

A straightforward scenario is to develop a subject-independent classification model based on the data from a subject population instead of to construct a subject-dependent model for each individual. This method works well under a common assumption that class distributions between individuals are similar to some extent. However, this may not be the case in real life, especially referring to the domains of cognitive and affective processing. Individuals may have different behavioral and/or (neuro) physiological responses to the same stimuli, which poses a significant challenge on developing an accurate generalized classifier that will fit all individuals. Such individual differences may explain a subject-independent classification model typically leading to marginal improvement or even considerable deterioration in performance as compared to a subject-dependent counterpart (Soleymani et al., 2012; Lin et al., 2014). In fact, the resultant emotion-related EEG features varied distinctly across individuals (Lin et al., 2010a, 2014; Zhu et al., 2015). Thus, the inter-subject approach needs to effectively cope with the individual difference to ensure the desired improvement.

Alternative to derive a general model, an emerging technology of transfer learning (TL; Pan and Yang, 2010) allows an individual to adapt his/her model to the data or information from other subjects selectively, so that the impact of the individual difference can be somehow alleviated. Recently, the TL has been successfully demonstrated in some BCI studies. Tu and Sun (2012) proposed a subject TL framework that employed an ensemble classification strategy that generated the final decision upon weighting to the model outputs of all subjects. Atyabi et al. (2013) specifically addressed the question of how to optimize the information from other subjects to the current subject played an important role in enhancing subject transfer. Kang et al. (2009) attempted to apply the subject transfer to a subject with fewer training samples to generate a better set of subject-specific features. The authors emphasized that the data of other subjects having data distributions similar to each other alleviated the individual difference. Wu et al. (2013) analogously reported that the subject who has small training samples could benefit from the data of other subjects having similar data distributions. Zheng et al. (2015) recently proposed a subject transfer framework to seek a set of low dimensional transfer components having much similar data distributions between subjects using transfer component analysis (TCA) and kernel principle component analysis (KPCA). The authors later (Zheng and Lu, 2016) further adopted transductive parameter transfer (TPT) proposed by Sangineto et al. (2014) to learn a subject-specific model without labeled data from other subjects. The TPT was found to outperform TCA and KPCA.

Based on the aforementioned evidence, the inter-subject TL featuring an effective selection on the subjects’ data and information should benefit the practicality of the EEG-based emotion classification. This study thus attempted to assess the efficacy of applying the inter-subject TL method to enhance the subject-specific emotion-classification model by reducing the amount of labeled data required for each individual. This study posed and empirically validated two hypotheses. First, it is reasonable to assume that some subjects might have difficulty in naturally engaging in an emotion-elicitation experiment within an unfamiliar laboratory setting. The recorded EEG dynamics of those subjects are less likely to be representative and informative about their emotional responses. This study thus hypothesized that the TL framework can considerably improve the classifiers of those poorly engaged subjects. This study named such a scenario as conditional TL (cTL). Second, although there are substantial individual differences in EEG signals (Lin et al., 2010a; Soleymani et al., 2012; Zhu et al., 2015), people may exhibit some common EEG signatures of the same emotions. This study thus further hypothesized that the improvement of classification performance obtained by the TL should positively correlate with the extent of the similarity of the subjects being included in TL. It is noted that even though recent works (Zheng et al., 2015; Zheng and Lu, 2016) studied the inter-subject TL framework in the EEG-based emotion classification, they did not address the subject transferability for transferring a new subject with respect to prior subjects, which could be a critical issue in real applications having a progressively growing subject repository. Once the two hypotheses of this study are validated, the proposed cTL could first identify the transferability of each individual who could benefit from the data from others for emotion classification and then selectively leverage the data from individuals having comparable EEG correlates of emotional responses for a positive transfer. The proposed cTL framework may not only shed light on the development of a more robust emotion-classification model using fewer labeled data, but may also facilitate the individualized calibration processing toward a real-life ABCI scenario.

## Materials and Methods

### EEG Dataset

This study adopted the Oscar soundtrack EEG dataset collected in Lin et al. (2010b) to test the proposed hypotheses and examine the efficacy of the cTL. The dataset consisted of 30-channel EEG signals collected from 26 healthy subjects while they were undergoing a music-listening and emotion label-tagging protocol. The experiment settings are briefly described as follows. The music protocol used 16 30-s music excerpts and intended to induce four emotion classes following the two-dimensional valence-arousal emotion model (Russell, 1980), including joy (positive valence and high arousal), anger (negative valence and high arousal), sadness (negative valence and low arousal) and pleasure (positive valence and low arousal). The experiment for each subject randomly separated the 16 excerpts (starting with 15-s silent rest) into four four-trial blocks with self-paced inter-block rest. Please refer to Lin et al. (2010b) for more details. Notably, since the four-class emotion samples were found highly imbalanced, this study opted to perform two-class valence (positive, i.e., joy and pleasure, vs. negative, i.e., anger and sadness) and arousal (high, i.e., joy and anger, vs. low, i.e., sadness and pleasure) classification tasks after regrouping the labels. Each of 26 subjects in the Oscar EEG dataset had 16 data pairs of 30-s EEG trials and two-class self-reported valence and arousal labels for analysis.

### EEG Feature Extraction, Selection and Classification

The raw 30-s 30-channel EEG signals of each trial were first transformed to the frequency domain using short-time Fourier transform with 50%-overlapping 1-s Hamming window. After applying a band-pass filter with a frequency range of 1–50 Hz, the estimated spectral time series of each channel was then grouped into five stereotyped frequency bands, including δ(1–3 Hz), θ(4–7 Hz), α(8–13 Hz), β(14–30 Hz) and γ(31–50 Hz). This study then adopted a feature type of differential laterality (DLAT; Lin et al., 2014) to reflect EEG spectral dynamics of emotional responses in a representation of hemispheric spectral asymmetry. Given 12 left-right symmetric channels (available in a 30-channel montage) and five frequency bands, DLAT generated a feature dimension of 60. Each spectral time series of DLAT was further divided by the mean power of its first 5 s for each trial followed by the gain model-based calibration method (Grandchamp and Delorme, 2011). Afterwards, the DLAT features were *z*-transformed across 16 trials to zero mean and unit variance for each subject.

Rather than utilizing the entire DLAT space, this study adopted a well-known feature selection method namely ReliefF (Robnik-Šikonja and Kononenko, 2003) to exploit a minimal yet optimal set of most informative features for each subject, which has been demonstrated effective in Jenke et al. (2014). The number of features with high ReliefF weight was determined based on the best training accuracy (described later). Lastly, this proof-of-concept study simply adopted a classifier of Gaussian Naïve Bayes (GNB) to model the data distributions belonging to positive vs. negative valence or high vs. low arousal classes. This study employed a Matlab (MathWorks, Inc., Natick, MA, USA) function called fitcnb() to use GNB modeling with default settings, which fitted the predictor distribution of each class with a Gaussian setting.

### Transfer Learning

TL is a machine-learning method with a perspective of providing a faster and better solution with less effort to recollect the needed training data and rebuild the model (Pan and Yang, 2010). In addition to its great progress in domains of document, speech, and image classification (Dai et al., 2007; Quattoni et al., 2008; Deng et al., 2013), recent neurophysiological studies (Kang et al., 2009; Tu and Sun, 2012; Atyabi et al., 2013; Wu et al., 2013) have also demonstrated the efficacy of the TL for improving the classification performance through using/learning the data/information from other individuals. Prior to depicting the proposed cTL framework in this study, the basic notations and definitions of TL are briefed as follows upon a TL review (Pan and Yang, 2010).

**Notation.** A domain *D* consists of two components: a feature space 𝒳 and a marginal probability distribution *P(X)*, in which *X* = {*x*_1_, … , *x_n_*} ∈ 𝒳. Given a specific domain *D* = {𝒳, *P(X)*}, a task consists of two components: a label space 𝒴 and an objective predictive function *f(•)*, denoted by *T*={𝒴, *f(•)*}, which can be learned from the training data pairs {*x_i_*, *y_i_*}, where *x_i_* ∈ 𝒳 and *y_i_* ∈ 𝒴. The *f(•)* can be used to predict the label of a new instance *x*, which can be rewritten by the probabilistic form of conditional probability distribution *P(Y|X)*. A task can then be defined as *T={*𝒴,*P(Y|X)}*. With the notations of domain and task, the TL is defined as following:

**Definition.** *Given a source domain *D_S_* and learning task *T_S_*, and a target domain *D_T_* and learning task *T_T_*, TL aims to help improve the learning of the target predictive function f(•) in *D_T_* using the knowledge in *D_S_* and *T_S_*, where *D_S_* ≠ *D_T_*, or *T_S_* ≠ *T_T_*.*

In the above definition, the condition *D_S_* ≠ *D_T_* refers to either 𝒳*_S_* ≠ 𝒳*_T_* or *P_S_*(*X*) ≠ *P_T_*(*X*), i.e., the source and target domains have different feature spaces or marginal probability distributions, whereas the condition *T_S_* ≠ *T_T_* means either 𝒴*_S_* ≠ 𝒴*_T_* or *P*(*Y_S_*|*X_S_*) ≠ *P*(*Y_T_*|*X_T_*), i.e., the source and target domains have different label spaces or conditional probability distributions. Note that when the target and source domains are the same, i.e., *D_S_* = *D_T_*, and their learning tasks are the same, i.e., *T_S_* = *T_T_*, the learning problem becomes a traditional machine-learning problem. If the TL improves the performance upon using solely *D_T_* and *T_T_*, the outcome is referred to a positive transfer. Otherwise, the TL deterioration leads to a negative transfer.

Referring to the targeted two-class valence and arousal emotion classification tasks using the EEG signals in this study, the domain refers to the EEG signals, whereas the task means the valence and arousal classification. As considering the transferring learning between subjects, the classification performance of a subject to be improved here is referred to the target subject (TS) from the target domain *D_T_*, whereas other existing subjects to be learned and transferred are called source subjects (SSs) from the source domain *D_S_*. In addition, due to individual differences in emotion perception and experience, subjects responded to the same emotion-induction materials may not only assign different emotion labels *Y*, but also may exhibit diverse spatio-spectral EEG dynamics *X*. The conditional probability distributions, i.e., *P*(*Y_S_*|*X_S_*) ≠ *P*(*Y_T_*|*X_T_*), between the TSs and SSs are thus different. On the other hand, the emotion-informative features are prone to vary from subject to subject (Lin et al., 2010b, 2014). This implies the existence of different feature spaces and corresponding marginal probability distributions of the TSs and SSs, i.e., 𝒳*_S_* ≠ 𝒳*_T_* and *P_S_*(*X*) ≠ *P_T_*(*X*). The aforementioned criteria accordingly explain the addressed EEG-based emotion classification task complied with the TL definition *D_S_* ≠ *D_T_* and *T_S_* ≠ *T_T_*.

### Conditional Transfer Learning

**Figure 1:**
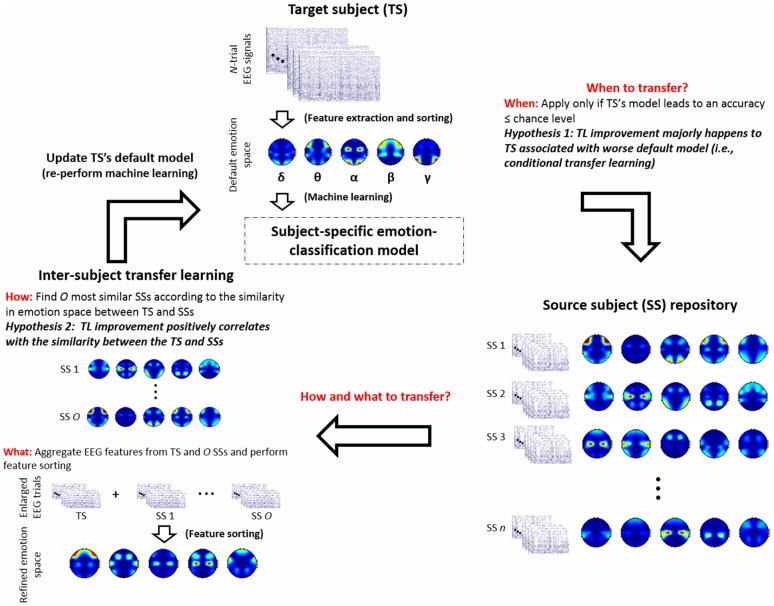
An overview of the proposed conditional transfer learning (cTL) framework with the emphasis on how to account for the two posed hypotheses regarding the individual differences in emotion perception (in italic fonts), including: (1) TL improvement majorly happens to target subjects (TSs) associated with worse default models; and (2) the extent of TL improvement positively correlates with the similarity of the TS and source subjects (SSs) being grouped, and how to address three leading issues to the success of positive TL (in red bold fonts), including: (1) when to transfer; (2) how to transfer; and 3) what to transfer. Note that *n* is the number of subjects treated as SSs (*n* = 25 in this study), whereas *O* is the number of similar subjects being selected from *n* SSs.

The inherent individual differences in emotion experience might lead to two following outcomes to a group of subjects after undergoing the same emotion experiment. On one hand, some subjects who might be less emotionally engaged in the experiment than others. Their data thus contained much less representative EEG correlates of emotional responses. On the other hand, despite the salient individual differences, neurophysiological responses for some subjects may exhibit some common EEG signatures about emotional responses. Such a group of similar subjects are presumably able to share the training data with each other for affective computation, as opposed to a group of dissimilar subjects. Accordingly, the present study posed two hypotheses for the inter-subject TL: (1) TL framework will benefit the classification models i.e., positive transfer, for the TSs whose models did not perform well based solely on their own data; and (2) the extent of the positive transfer tends to positively correlate with how similar the SSs being included to the training data for the TS. Figure 1 gives an overview of the proposed cTL framework regarding how to deal with the issues to the success of positive TL (Pan and Yang, 2010), including: (1) when to transfer; (2) how to transfer; and (3) what to transfer.

1. When to transfer
  Brute-force transfer, i.e., blindly applying TL, sometimes may cause a negative transfer as the target and source domains apparently differ (Pan and Yang, 2010). Defining the transferability from the source to target domain of interest is thus imperative. As mentioned previously, due to the individual differences, subject-specific emotion-classification performance may vary widely from subject to subject (Lin et al., 2014). Some of them were marginally above or even below chance level, i.e., random guessing. This also in part explains the previously reported classification performance of a subject population was not satisfactorily high against chance level (Koelstra et al., 2012; Soleymani et al., 2012; Lin et al., 2014). This outcome suggested us to treat the chance level as a benchmark inferring the TL transferability to a given TS. In this regard, this study hypothesized that the TL method will benefit the TSs whose default classification models, i.e., trained by their own data, merely achieve a below-chance-level accuracy (i.e., 50% in a two-class classification problem). On the contrary, brute-force transfer for the good subjects (default accuracy >50%) will likely cause a negative transfer. Such a hypothesis-orientated TL scenario was named cTL as opposed to the standard TL that applies transferring to all subjects.
2. How to transfer
  Most machine-learning methods can work well under the assumption that the training and test data are drawn from the same distribution and feature space. It presumably also applies to the TL framework. As such, sharing the data or information between subjects who have comparable neurophysiological patterns theoretically results in prominent improvement as opposed to that between dissimilar subjects. To this end, this study characterized the inter-subject similarity based on their ReliefF-sorted emotion-relevant feature spaces. The similarity was defined be the value of the Pearson’s correlation coefficient. That is, the larger the correlation coefficient value is, the more similar the subjects are. Lastly, the TL framework included a group of most similar SSs as the training data to develop a new emotion-classification model for the given TS.
3. What to transfer
  A natural next question is which part of knowledge from the selected SSs can be extracted to transfer for the TS. This study concatenated the labeled data of the selected SSs and TS together to develop a more generalized yet informative feature space. The augmented training data and refined feature space was then used to re-build the emotion-classification model for the TS.

### Validation of the Proposed Conditional Transfer Learning Framework

Each of the included 26 subjects was in turn treated as a TS to test the proposed cTL framework with the remaining 25 subjects regarded as SSs. Furthermore, the leave-trial-out (LTO) validation method was applied to the TS’s data trials to yield the subject-dependent classification accuracy. In this way, the TS’s default performance refers to the LTO accuracy solely based on their own data, i.e., without TL, whereas the TL-improved performance refers to the result obtained by leveraging the TS’s 15 out of 16 trials with all of the trials from the selected SSs to train the model and test it against the left-out trial from the TS in each LTO repetition. The information of a test trial of a given TS was entirely disjointed from the optimization of a classification model as well as the selection of transferrable SSs, which complied with a realistic BCI validation regime. The TL steps in each LTO repetition are described as follows:

1. *Calculate the similarity between the TS and SSs*.
  Using 15 training trials to form the TS’s feature space using ReliefF and calculating correlation coefficient values between this space to the space of each of n SSs (*n* = 25 in this study).
2. *Transfer the data of the most similar SS(s) to the TS*.
  Merging the 15 training trials of the TS with the data trials of the *O* most similar SS(s), where *O* is the number of subjects being selected, and then using the augmented dataset (15 + *O* ×16 trials) to re-form the TS’s feature space using ReliefF.
3. *Optimize the feature space of the TS*.
  In order to discard the impact of the class imbalance problem during classifier modeling, this step randomly selected class-balanced samples on the augmented training trials with 500 repetitions in an attempt to yield an optimal yet fair training model. Each repetition applied 5-fold cross-validation and add-one-feature-in, i.e., adding one feature with a high ReliefF score at a time, methods to exploit an optimal feature subspace with the maximal training accuracy.
4. *Train and test the TL-refined model of the TS*.
  The GNB model was re-trained with the augmented and feature space-optimized data trials and was tested against the disjointed left-out trial of the TS.

To demonstrate the validity of the posed cTL scenario, this study also employed another two TL scenarios, namely routine TL (rTL) and optimal TL (oTL), as comparative benchmarks. The rTL is the conventional framework that equally applies TL to each TS without evaluating their transferability, whereas the oTL enforces a TS’s default model adapting to the TL-refined counterpart as long as a positive transfer occurs. The oTL is a heuristic-optimization procedure that allows every TS have a chance to improve their default model and thereby led to the best performance.

## Results

**Figure 2:**
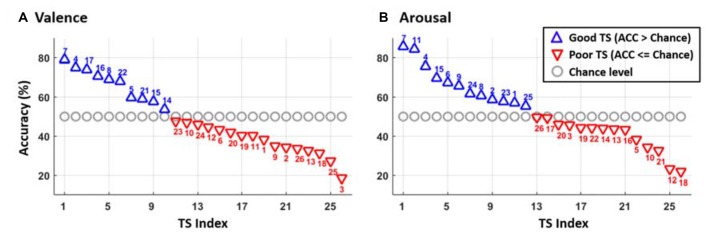
The two-class classification accuracy of **(A)** valence and **(B)** arousal emotions for each of 26 TSs sorted by their performance. The blue upward-pointing triangles represent the good TS group with the above-chance level accuracy, whereas the red downward-pointing triangles indicate the poor TS group corresponding to the accuracy near or below chance level. The gray circles show the chance level of 50% for two classes. The numbers above/under the triangles indicate the TS label.

Figure 2 portrays the subject-specific two-class valence and arousal classification performance solely using the data from each TS. As can be seen, 16 of 26 TSs had accuracy below 50% (red downward-pointing triangles) in the valence classification, whereas the arousal classification returned slightly fewer poor TSs of 14. Particularly, eight TSs (e.g., 3, 10, 12, 13, 18, 19, 20 and 26) failed to provide above-chance accuracy in both emotion categories. On average, the 26 TSs obtained the accuracies of 48.58 ± 16.44% but varied widely (max: 85.43%, min: 21.79%) and 52.14 ± 16.48% (max: 78.68%, min: 35.06%) in valence and arousal classification, respectively.

**Figure 3:**
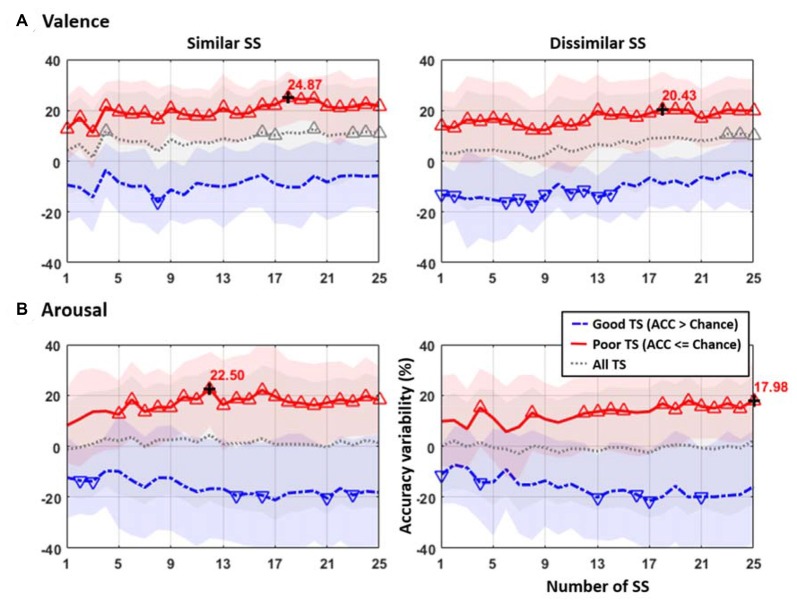
The accuracy variability obtained by TL to the TSs that incorporated different numbers of similar or dissimilar SSs with reference to their default classification accuracy of **(A)** valence and **(B)** arousal emotions. The results were categorized into three TS groups according to their default performance, including Good (default accuracy (ACC) > 50%, with blue dash-dot line), Poor (default accuracy ≤ 50%, with red solid line), and All (both Good and Poor TSs, with gray dashed line). The colored, shaded areas along the profile show the standard deviation of the classification performance. The upward-pointing and downward-pointing triangles represent the accuracy improvement and deterioration significantly varied from zero using a one-sample *t*-test with *p* < 0.01, respectively. The maximal improvement was marked by the black cross. The number of SS in *x-axis* refers to the number of SSs being selected for TL to the TS, whereas the accuracy variability in *y-axis* indicates the improvement or deterioration in classification accuracy after TL was applied with respect to the default performance of the TS.

Figure 3 presents the accuracy variability obtained by TL to the TSs that incorporated different numbers of similar or dissimilar SSs from 1 to 25 with respect to their default performance. For both valence and arousal classifications, only the poor TS group (red solid profile) returned salient improvements after TL, whereas the good TS group (blue dash-dot profile) conversely led to deteriorated performance. The All TS group (gray dotted profile) that did not separate TSs by their default performance returned minor improvement in valence but not in arousal category. Furthermore, the TL based on similar and dissimilar SSs resulted in distinct outcomes. For the valence classification, the use of a group of 18 similar or dissimilar SSs resulted in maximal improvements (similar: 24.87% vs. dissimilar: 20.43%). Nevertheless, the poor TS group distinctly benefitted from TL in the arousal classification. Using only 12 similar SSs promptly resulted in a maximal improvement of 22.50%, which was almost double to that using the group of 12 dissimilar SSs (12.95%). The scenario of using dissimilar SSs in arousal state instead returned a slow increment to the maximum of 17.98% as all 25 SSs were included. Lastly, for both emotion categories, TL based on data from all 25 SSs was not necessarily leading to a maximal improvement for most comparative conditions.

**Figure 4:**
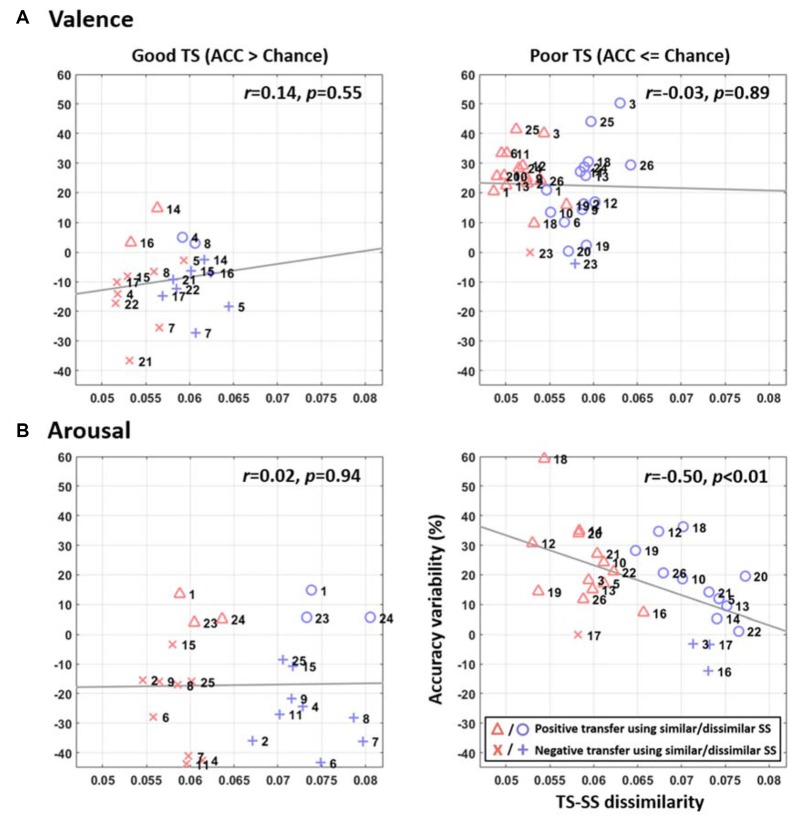
The relationship between accuracy variability by TL and inter-subject similarity for **(A)** valence and **(B)** arousal emotions for each TS, which was assessed based on the resultant optimal TL scenarios for valence (using 18 similar SSs, SSs) and arousal (using 12 similar SSs) categories (see Figure 3). The triangles and circles represent the positive transfer, i.e., improved performance, whereas the crosses represent the negative transfer, i.e., deteriorated performance. The symbols in red and blue indicate the inclusions of similar and dissimilar SSs, respectively. The gray lines depict the linear relationship between the accuracy improvements over TS-SS dissimilarity assessed by linear regression analysis. The TS-SS dissimilarity in *x*-axis shows the standard deviation of the absolute correlation coefficients of the available TS-SS pairs (the larger the value was, the more the dissimilar subjects were being included for TL), whereas the accuracy variability in *y-axis* indicates the improvement or deterioration in classification accuracy after TL was applied with respect to the default performance of the TS.

Figure 4 further shows the relationship between accuracy improvement by TL and inter-subject similarity for each TS based on the resultant optimal TL parameters of 18 and 12 similar SSs for valence and arousal categories (i.e., could lead to maximum TL improvement as shown in Figure 3). Several results are worth mentioning here. First, pooling similar SSs as opposed to dissimilar SSs for a given TS typically resulted in smaller TS-SS dissimilarity (red triangles and crosses vs. blue circles and crosses). The TS-SS dissimilarity in the valence plots tended to vary within a narrower range of 0.045–0.065 (Figure 4A) than a range of 0.053–0.081 in the arousal plots (Figure 4B). Second, the TL consistently resulted in positive transfer (triangles and circles) for the poor TSs, but generally led to a negative transfer (crosses) for the good TSs for both emotion categories. By leveraging similar SSs, 15 of 16 and 13 of 14 defined poor TSs obtained improvements in valence and arousal classification, respectively. Lastly, further comparing the inclusions of similar vs. dissimilar SSs, TL revealed a salient negative correlation between accuracy improvement and TS-SS dissimilarity in arousal classification (*r* = −0.5, *p* < 0.01), but not in the valence classification (*r* = −0.03, *p* = 0.89).

**Figure 5:**
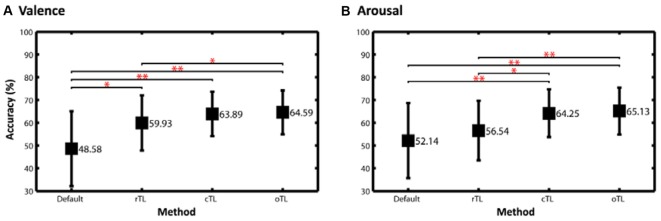
The two-class classification accuracy of **(A)** valence and **(B)** arousal emotions using different scenarios, including default, a routine TL (rTL), the proposed cTL and an optimal TL model (oTL). The rTL was the conventional framework routinely applying TL to each TS regardless of their default performance, whereas the oTL enforced each TS’s default model adapting to the TL-refined one as long as a positive transfer occurred. The statistical significance of differences in performance between two scenarios was assessed by paired two-sample *t*-tests (*p < 0.05, **p < 0.01).

Figure 5 compares the classification performance using different scenarios, including default learning (without TL), a routine transfer learning (rTL), the proposed cTL, and an optimal TL model (oTL). The rTL was the conventional framework routinely applying TL to each TS regardless of their default performance, whereas the oTL enforced the TS’s default model adapting to the TL-refined counterpart as long as positive transfer occurred, which allowed every TS have a chance to improve their default model and thereby led to the best performance. As can be seen, the rTL resulted in a performance improvement of ~11% (*p* < 0.05) for valence and 4% for arousal classification upon the default performance. The proposed cTL made the improvement much salient, which were almost up to 15% and 12% for valence and arousal categories (*p* < 0.01). In other words, the cTL outperformed the rTL by ~4% and ~8% (*p* < 0.05) for valence and arousal classifications, respectively. Most importantly, for both emotion categories the cTL was comparable (*p* > 0.05) to the oTL that alternatively adopted the TL-refined model if a positive transfer was desirable.

## Discussion

### Defining Subject Transferability for Positive Transfer

For the interpretation of when to transfer the following results can be considered. Without using TL, the average classification accuracies of valence (48.58 ± 16.44%) and arousal (52.14 ± 16.48%) for the 26 subjects in this study (see Figure 5) were found to be near the chance level (50%). In fact, as shown in Figure 2, the classification accuracy in some subjects exceeded 70%, but in some subjects had the sub-30% accuracy. This result might be attributed to inevitable individual differences (as is evident from a high standard deviation ~16%). We speculated that some participants may have difficulty in emotionally engaging in the designed experiment within an unfamiliar laboratory setting, especially wearing an awkward EEG headset. This study was thus motivated to propose the conditional TL under the assumption that the TL would profit subjects with low default performance rather than those with relatively good performance. This study intuitively adopted the criterion of the default performance above or below chance level, i.e., 50%, to infer the TL transferability of a given TS. As shown in Figures 3, 4, brute-force transfer to the good TS group typically resulted in a negative transfer. Thus, when the proposed conditional TL was used on the poor TS group only, the obtained improvement in performance was better than that using the routine TL (see Figure 5). The study results not only empirically proved the feasibility of using a simple criterion based on the default-model performance, but also supported that exploring “when to transfer” is an imperative step (Pan and Yang, 2010) to the success of a positive transfer. In the future, the generalizability of the inclusion criterion based on the chance level of the number of tasks to be classified can be further tested on other emotion datasets (Koelstra et al., 2012; Lin et al., 2015a).

Furthermore, as inspecting the cTL-oTL comparison (see Figure 5), one might argue that the oTL scenario, which adapted the default model to the TL-augmented counterpart as long as the positive transfer occurred, could be an alternative remedy without considering the issue of subject transferability and the risk of a negative transfer. However, the oTL scenario is a heuristic approach and only feasible for a small subject repository. Once the size of the repository increases, applying brute-force transfer for each subject inevitably makes a heavy burden on the limited computation resources and unnecessary computations since the negative transfer may occur frequently. Thus, this study instead proposed the cTL to address the issue “when to transfer” for avoiding negative transfer as well as to reduce computational complexity.

### Grouping Similar vs. Dissimilar Source Subjects for Positive Transfer

This study evidently validated the posed hypothesis that pooling a group of similar SSs may lead to a noticeable augmentation as opposed to a group of dissimilar SSs. First, the TS’s emotion-classification model obtained an immediate improvement by leveraging 5~10 similar SSs and afterwards reached to a maximal improvement by recruiting more similar SSs involved (valence: 18 SSs, arousal: 12 SSs, see Figure 3). This trend did not exactly replicate in the case of recruiting the same number of dissimilar SSs. Especially, the arousal category showed that the TL augmentation for the poor TS group significantly correlated with the similarity of the SSs to be transferred (see Figure 4). Regarding the less distinguishable contributions made by using similar and dissimilar SSs in the valence category, the reason was very likely attributed to that the included 26 subjects possessed a relatively similar DLAT feature space in valence compared to in arousal, so that pooling either similar or dissimilar SSs for valence transferring resulted in a narrower TS-SS dissimilarity range (0.045–0.065, see Figure 4). This indicated that the differences between similar and dissimilar subjects in the valence category were insignificant. As such, pooling data from other subjects (either similar or dissimilar SSs) would result in a positive transfer for the valence classification. Previous BCI studies analogously reported the efficacy of adopting similar subjects for leveraging the information between subjects in an attempt to boost the task performance (Kang et al., 2009; Samek et al., 2013; Wu et al., 2013).

Second, aggregating all available subjects together did not absolutely guarantee a maximal enhancement in performance either for the case of using similar or dissimilar SSs. Due to the individual differences, aggregating all available SSs may make the class distributions much overlapped or even conflicted. This may partially support the finding in an earlier subject-independent emotion classification study (Soleymani et al., 2012; Lin et al., 2014) and a study comparing subject-dependent vs. subject-independent manner using other biopsychological signals (Böck et al., 2012).

The objective of this study was to assess the feasibility of the TL in the EEG-based emotion-classification. This proof-of-concept study has not fully explored an optimal threshold for the inter-subject similarity, i.e., TS-SS dissimilarity value, for choosing the number of similar SSs to be grouped and transferred to each TS. This study leaves this issue to a separate study with a larger subject population in the future. Another tentative direction is to incorporate other EEG emotion datasets having distinct groups of subjects, such as an open-access dataset of DEAP (Koelstra et al., 2012) or the dataset collected in Lin et al. (2015a), which can save considerable time and labor efforts for increasing the subject population in a single experiment. However, it might introduce a new challenge in how to reasonably cope with (but may not limit to) different settings of electrode montages that used in data recordings, and different materials for emotion elicitation in each dataset.

### Comparing the Obtained Classification Results with Previous Works

This study next compared the obtained two-class emotion-classification performance without and with TL methodology to the recent works that also addressed two-class valence and arousal classification tasks using limited data trials (Koelstra et al., 2012; Koelstra and Patras, 2013; Lin et al., 2014). Without using TL, the default valence and arousal of this study (see Figure 5) were found to be lower than the reported results of 57.6% for valence and 62% for arousal (see their EEG modality in Table 7 Koelstra et al. (2012)). Once the default model augmented by the proposed cTL framework, the classification performance reached ~64% for both categories. However, such TL-based augmentation remained worse than the reported results in (Koelstra and Patras, 2013; valence: 70%, arousal: 67.5%, see their EEG modality and RFE feature type in Table 3) and the results of ~70% for valence and arousal classifications (Lin et al., 2014; see their DLAT feature type in Figure 2). It is worth noting that using different machine learning frameworks (feature extraction, selection and classification), applying different validation methods (offline or online), and/or conducting different emotion datasets all potentially lead to variations in default performance of each individual. Instead of comparing with the default performance of previous works, this study stressed out that the proposed cTL led to a prominent improvement by selectively leveraging data from other subjects, i.e., without increasing the labeled training data from each individual, which is expected more prominent in an ever-growing data repository in real-life applications.

## Conclusion

This study proposed a scenario namely conditional TL to first assess a subject’s transferability for improving classification performance and then leverage the data from other subjects having informative yet comparable feature spaces. Upon the validation on a 26-subject EEG dataset, the study results empirically showed that the proposed cTL framework led to a maximal improvement of ~15% and ~12% by recruiting data from 18 and 12 subjects who had similar EEG signatures for two-class valence and arousal classification, respectively, with reference to the default performance solely using the subject-specific data. The cTL-augmented performance also distinctly outperformed that using the conventional TL (blindly applying TL to each subject without considering the risk of deterioration in performance) by ~4% and ~8% for valence and arousal categories.

## Ethics Statement

The study was conducted in accordance with the Declaration of Helsinki and approved by UCSD Human Research Protections Program.

## Author Contributions

Y-PL conceived and performed the data analysis; Y-PL and T-PJ wrote the article.

## Conflict of Interest Statement

The authors declare that the research was conducted in the absence of any commercial or financial relationships that could be construed as a potential conflict of interest.
